# An Atypical Presentation of Ecthyma Gangrenosum in a Patient With Waldenström Macroglobulinemia

**DOI:** 10.7759/cureus.103263

**Published:** 2026-02-09

**Authors:** Sarah R Eggert-Cichocki, Madison S Meyer, Anastasia Goldbaum, Khalid Zakaria

**Affiliations:** 1 Internal Medicine, Wayne State University School of Medicine, Detroit, USA; 2 Internal Medicine, Henry Ford Health System, Novi, USA

**Keywords:** atypical presentation, concurrent chemotherapy, ecthyma gangrenosum (eg), immuno-compromise, pseudomonas infections, waldenstrom's macroglobulinemia

## Abstract

Ecthyma gangrenosum (EG) is a relatively rare cutaneous infection that is most commonly caused by *Pseudomonas aeruginosa (P. aeruginosa)*. EG predominantly affects immunocompromised individuals and can be categorized into bacteremic and non-bacteremic types. Despite this distinction and clinical evidence supporting the existence of the non-bacteremic type of EG, the long-held belief that bacteremia must be present for the diagnosis of EG persists. This report describes a case of a 78-year-old Caucasian male with Waldenström macroglobulinemia (WM) undergoing chemotherapy, who presented with a non-tender, necrotic, gangrenous ulcer on the left side of his groin. Physical examination revealed a sharply demarcated black eschar measuring 3 cm in diameter with an erythematous border. Laboratory results showed a white blood cell count of 1.34 K/mcL, red blood cell count of 4.07 million/µL, and platelet count of 126 K/mcL, indicating marked pancytopenia. Blood cultures obtained on the day of admission were negative. Tissue cultures from a punch biopsy were positive for *P. aeruginosa* and *Enterococcus faecalis (E. faecalis)*. Initial empiric treatment consisted of intravenous cefepime and vancomycin. Full surgical debridement was performed on day four. Following the procedure, the patient was discharged the same day on amoxicillin-clavulanate and ciprofloxacin to be taken for 10 days and was instructed to follow up with wound care. The lesion site showed improvement with the antibiotic regimen, both before and after debridement.

EG can manifest without bacteremia, especially in immunosuppressed individuals with environmental risk factors. In this case, the patient’s history of WM, use of the chemotherapeutic agent zanubrutinib, and neutropenia compromised innate host defenses. These factors, combined with a recent hospitalization that included the administration of broad-spectrum antibiotics, created a favorable environment for bacterial growth. This report serves as a reminder that prompt initiation of empiric antibiotics, timely biopsy of the lesion, and subsequent tissue cultures to determine bacterial susceptibility are essential for a favorable outcome when non-bacteremic EG is suspected.

## Introduction

Ecthyma gangrenosum (EG) is a relatively rare cutaneous infection that predominantly affects immunocompromised individuals. In these patients, approximately 62% to 75% have an underlying immunodeficiency [1]. EG typically begins as a pustule that develops into a hard, crusted lesion overlying a necrotic ulcer, surrounded by an erythematous border [1]. The most frequently identified organism in EG is *Pseudomonas aeruginosa (P. aeruginosa)*, a non-lactose fermenting, gram-negative aerobic coccobacillus. Other potential bacterial causes of EG include *Escherichia coli (E. coli), Klebsiella pneumoniae (K. pneumoniae), Streptococcus pyogenes (S. pyogenes)*, and *Citrobacter freundii (C. freundii)*, among others [1]. EG was first reported in 1897 by Dr. Lewellys Barker, in which case it presented as *Pseudomonas* septicemia [1]. Traditionally, the clinical picture of EG has been considered a necrotic lesion preceded by *Pseudomonas* septicemia. However, since the 1980s, evidence has supported the existence of both bacteremic and non-bacteremic forms of EG.

A 2001 case report, for instance, described a patient with acute myelogenous leukemia who developed a large, solitary necrotic ulcer that tested positive for *P. aeruginosa* without preceding bacteremia [2]. More recently, a 2020 case report detailed a single EG lesion in a 54-year-old woman with a heart transplant, which also presented without bacteremia [3]. These cases, along with a recent meta-analysis of 167 case reports that found only 55.8% involved *Pseudomonas* septicemia, suggest that the absence of bacteremia is not uncommon. Furthermore, this analysis found that the triad of a *P. aeruginosa* lesion, *Pseudomonas* sepsis, and immunocompromised status was present in only 19.2% of reported cases [4]. The pathophysiology of non-bacteremic *P. aeruginosa* EG is thought to occur via direct inoculation of the affected site, with skin and vascular destruction caused by virulence toxins and enzymes released by the bacteria, including exotoxin A, elastase, and phospholipase C [1,5].

Waldenström macroglobulinemia (WM), also known as lymphoplasmacytic lymphoma, is a rare, slow-growing B-cell lymphoma characterized by the overproduction of an abnormal immunoglobulin M (IgM) monoclonal protein [6]. Of all hematologic malignancies, WM represents only approximately 2% [6]. The disease typically presents in males in the seventh or eighth decade of life, with most individuals surviving seven to eight years after diagnosis [6]. As a B-cell lymphoma, B symptoms such as night sweats, fever, and weight loss are common. Additionally, bone marrow infiltration is frequently present, leading to fatigue and/or weakness secondary to pancytopenia [6]. The criteria for diagnosing WM are as follows: 1) presence of IgM monoclonal gammopathy; 2) infiltration of bone marrow by small lymphocytes showing plasmacytoid or plasma cell differentiation; 3) bone marrow infiltration with an intertrabecular pattern; and 4) an immunophenotype supportive of WM, including surface IgM+, CD19+, CD20+, CD22+, CD25+, CD27+, FMC7+, variable CD5, CD10-, CD23-, CD103-, and CD108- [6]. This report details the case of a patient with WM who, while receiving chemotherapy, developed a necrotic EG lesion despite the absence of classic systemic signs of infection or bacteremia.

## Case presentation

The patient was a 78-year-old male admitted for a necrotic and gangrenous ulcer in the left groin. He had reportedly noticed the lesion approximately 10 days earlier. He denied any preceding erythema at the area of the lesion, systemic symptoms, or any trauma to the area. He stated that he had last taken doxycycline five days before presentation for a respiratory tract infection and reported that the lesion had been present before the onset of pulmonary symptoms. No respiratory symptoms were present at the time of presentation. Regarding his medical history, the patient had been diagnosed with WM the previous year and had been receiving zanubrutinib chemotherapy for the preceding three months. Chemotherapy had been held during hospitalization. Furthermore, approximately 10 months earlier, he had experienced *E. coli* sepsis, complicated by necrotizing fasciitis involving the left lower extremity and a *Candida* infection of stage II pressure ulcers in his groin. He had required a prolonged course of intravenous (IV) cefepime during that admission and also received metronidazole and fluconazole. He had additionally received Neupogen to bolster his neutrophil count and had subsequently shown significant improvement. He had been discharged after 15 days and prescribed a two-week course of amoxicillin-clavulanate upon discharge.

**Figure 1:**
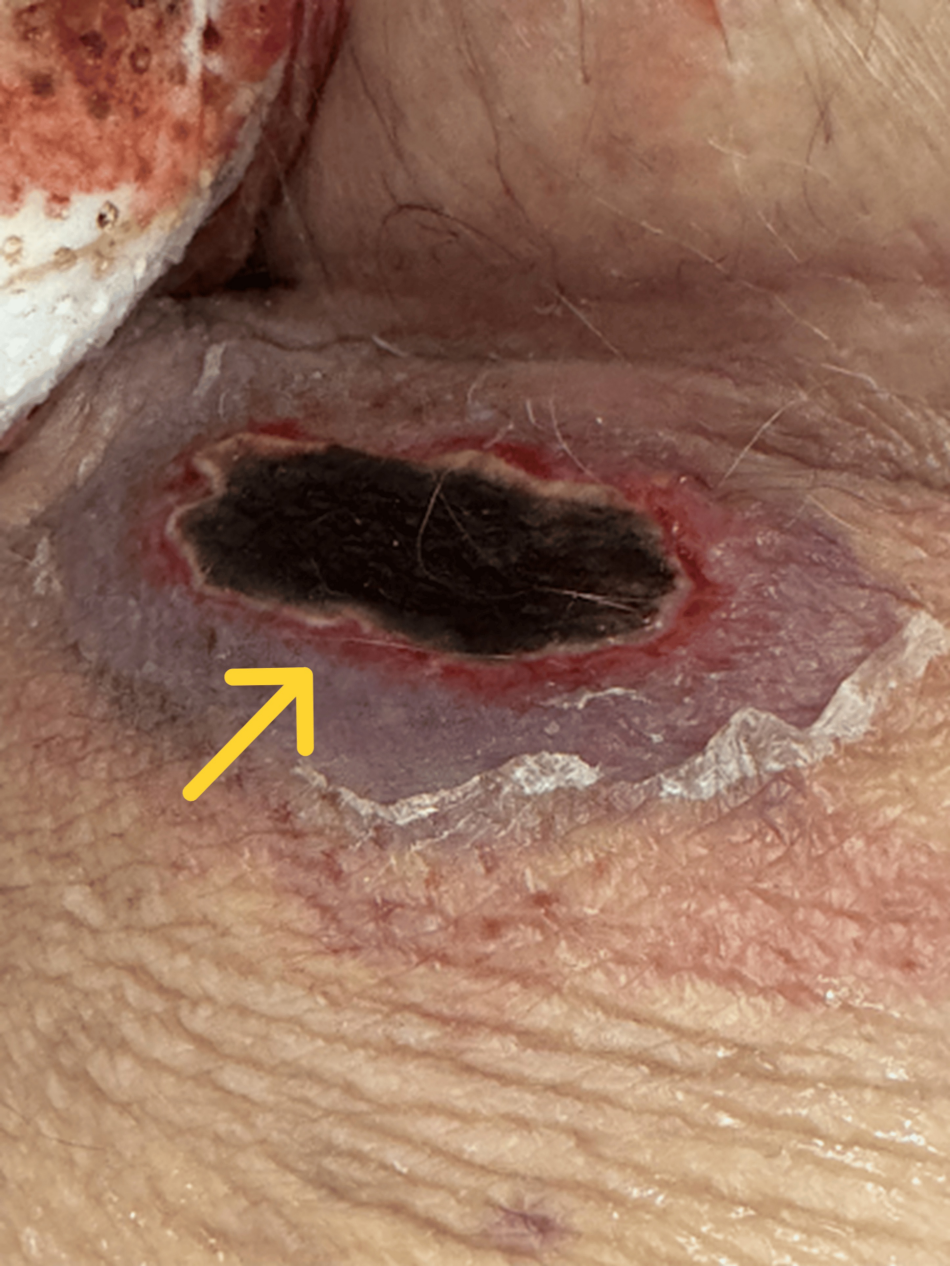
Ecthyma gangrenosum lesion A close-up view of the patient’s lesion. It is notable for its central black eschar and erythematous rim (rim highlighted by yellow arrow), signifying a gangrenous and necrotic process; this is consistent with the classic appearance of ecthyma gangrenosum

Physical examination revealed a black eschar measuring approximately 3 cm in diameter, with surrounding erythema as shown in Figure 1. The patient was afebrile, and no lymphadenopathy was noted. Laboratory findings revealed pancytopenia and a decreased absolute neutrophil count (ANC), which are classically expected with WM (Table 1). Blood cultures were collected on the day of admission. These cultures remained negative after five days. Beginning on hospital day one, the patient received empiric treatment with IV cefepime and IV vancomycin throughout his admission. The medical team was initially skeptical that the lesion could be due to ecthyma gangrenosum, as the patient did not exhibit systemic symptoms. The dermatology team was consulted, and suspected that the lesion was more likely a malignancy due to the absence of clinical signs of septicemia.

**Table 1:** Laboratory results From admission (day 1) to discharge (day 4), the patient’s laboratory results consistently demonstrated pancytopenia. White blood cell (WBC), red blood cell (RBC), and platelet counts were all below their respective reference ranges. The patient also had a persistently decreased absolute neutrophil count (ANC) throughout the hospital course. Laboratory studies were not obtained on hospital day 3

Variables	Day 1	Day 2	Day 4	Reference ranges
WBC, K/mcL	1.34	1.36	1.56	4–10
RBC, million/µL	4.07	3.98	3.71	Males: 4.7–6.1
Platelets, K/mcL	126	134	116	150–450
ANC, K/mcL	0.61	0.35	0.96	1.5–7.7

However, on hospital day three, an excisional punch biopsy was performed. Tissue culture was positive for *P. aeruginosa *and *Enterococcus faecalis (E. faecalis)*. On hospital day four, complete debridement of the wound was performed, and the patient was subsequently discharged home. Throughout his admission, the patient repeatedly denied systemic symptoms and remained hemodynamically stable. Moreover, the lesion showed improvement following initiation of antibiotic therapy, as the surrounding erythema decreased. The patient was discharged with a prescription for amoxicillin-clavulanate and ciprofloxacin for a 10-day course. After discharge, he was followed in the outpatient wound care center, where his daily regimen included Iodosorb gel and bandage changes. The wound was noted by the infectious disease team to be nearly completely healed 30 days later, when the patient was re-admitted to the hospital for a separate illness.

## Discussion

This patient’s clinical picture is consistent with primary cutaneous EG without bacteremia. The firm, oval, approximately 3 cm necrotic plaque located in the groin progressively enlarged over 10 days. The lesion presented as a nontender, sharply demarcated black eschar with a subtle erythematous rim, as shown in Figure 1. On admission, the patient was normothermic (96.7 °F) with negative blood cultures in the setting of leukopenia (WBC 1.34 × 10³/µL). This initially supported a localized vasculotropic process rather than a septicemic disease. *P. aeruginosa* was isolated from the tissue culture and confirmed the diagnosis. The presence of concomitant *E. faecalis* is consistent with superficial colonization in an intertriginous site. WM), Bruton tyrosine kinase (BTK) inhibition with zanubrutinib, and neutropenia may have acted synergistically to impair innate host defenses [6,7]. In addition, the occluded groin environment and recent doxycycline exposure likely favored pseudomonal proliferation with subsequent vascular invasion and dermal infarction [8].

The combination of host and environmental factors enabled *P. aeruginosa* invasion of cutaneous blood vessels [9,10]. WM can cause microvascular perfusion defects through IgM accumulation and is associated with dysfunction of the humoral immune system [11,12]. Further, zanubrutinib inhibited BTK-dependent neutrophil innate effector functions [13]. The intertriginous groin area is prone to friction and moisture with limited air circulation, leading to fissures and secondary bacterial infection [14]. Recent hospitalization for necrotizing fasciitis and broad-spectrum antibiotics likely affected both skin and gastrointestinal flora, which may have increased the potential for *Pseudomonas* colonization. The preadmission course of doxycycline was not antipseudomonal and provided no coverage for the lesion.

The treatment approach was individualized based on the integration of initial biopsy results with broad-spectrum empiric therapy. Initial treatment consisted of intravenous vancomycin in combination with intravenous cefepime, providing antipseudomonal coverage. As the patient improved on this antibiotic regimen, this response supported the diagnosis of vascular gram-negative invasion. Oral ciprofloxacin was later selected as the preferred agent against *P. aeruginosa* during the patient’s clinical course [15]. Addressing the enterococcal infection with amoxicillin-clavulanate was an additional key to his recovery. Zanubrutinib was held temporarily during the acute infection to allow host defenses to reconstitute with reduced immunosuppressive pressure. The small, well-demarcated eschar remained stable, without evidence of fluctuance or deep fascial involvement, and was managed with iodine-based gel wound care alone, without any need for surgical debridement. The consequences of surgery would likely have been more detrimental than beneficial by increasing tissue loss in this profoundly leukopenic patient.

This report underscores several issues that are diagnostically and therapeutically relevant. Negative blood cultures alone did not exclude EG. Reliance on systemic microbiology may have delayed appropriate therapy. The afebrile, hemodynamically stable presentation in this elderly patient demonstrates how advanced age and cytopenias can obscure the severity of these vasculopathic skin infections. The anatomic distribution of this infection in the groin, which is unusual for EG, may be misattributed to irritant dermatitis or ecchymosis in the absence of pain and systemic illness. In this setting, an expedited tissue diagnosis can prevent diagnostic drift. Finally, infection of the ulcer with a second organism underscores the importance of interpreting microbiology in both anatomic and pathophysiologic contexts, which supports prioritizing therapy against the vasculotropic pathogen while adding adjunctive coverage only when it is likely to alter the outcome of treatment.

## Conclusions

This report adds to the growing body of literature showing that EG can manifest without bacteremia, especially in immunosuppressed individuals with environmental risk factors. In the case presented, WM, use of the chemotherapeutic agent zanubrutinib, and neutropenia likely impaired innate host defenses. Furthermore, a recent hospitalization that included the administration of broad-spectrum antibiotics may have created a favorable environment for bacterial growth. Together, these factors likely contributed to the development of EG in the absence of classic systemic symptoms. The proposed mechanism of infection without bacteremia is direct inoculation at the affected site, leading to skin and vascular destruction caused by virulence toxins and enzymes released by the bacteria and subsequent lesion formation. If nonbacteremic EG is suspected, prompt initiation of broad-spectrum antibiotics and timely biopsy of the lesion, with subsequent transition to pathogen-specific antibiotics, is essential for a favorable outcome. Overall, this report reinforces the need to maintain a high index of suspicion for EG in immunocompromised individuals who are not bacteremic.

## References

[1] Shah M, Crane JS (2025). Ecthyma Gangrenosum. https://www.ncbi.nlm.nih.gov/books/NBK534777/.

[2] Song WK, Kim YC, Park HJ, Cinn YW (2001). Ecthyma gangrenosum without bacteraemia in a leukaemic patient. Clin Exp Dermatol.

[3] Hamed A, Niehaus AG, Bosshardt Hughes O, Jakharia N (2020). Ecthyma gangrenosum without bacteremia in a 54-year-old woman with heart transplant. Transpl Infect Dis.

[4] Vaiman M, Lazarovitch T, Heller L, Lotan G (2015). Ecthyma gangrenosum and ecthyma-like lesions: review article. Eur J Clin Microbiol Infect Dis.

[5] Vaiman M, Lasarovitch T, Heller L, Lotan G (2015). Ecthyma gangrenosum versus ecthyma-like lesions: should we separate these conditions?. Acta Dermatovenerol Alp Pannonica Adriat.

[6] Kaseb H, Gonzalez-Mosquera LF, Parsi M, Mewawalla P (2025). Lymphoplasmacytic Lymphoma. https://www.ncbi.nlm.nih.gov/books/NBK513356/.

[7] Desai JV, Zarakas MA, Wishart AL (2024). BTK drives neutrophil activation for sterilizing antifungal immunity. J Clin Invest.

[8] Grossman TH (2016). Tetracycline antibiotics and resistance. Cold Spring Harb Perspect Med.

[9] Sutherland CA, Quest TL, Wanat KA (2023). Ecthyma gangrenosum. IDCases.

[10] Qin S, Xiao W, Zhou C (2022). Pseudomonas aeruginosa: pathogenesis, virulence factors, antibiotic resistance, interaction with host, technology advances and emerging therapeutics. Signal Transduct Target Ther.

[11] Bibas M, Sarosiek S, Castillo JJ (2024). Waldenström Macroglobulinemia - a state-of-the-art review: part 1: epidemiology, pathogenesis, clinicopathologic characteristics, differential diagnosis, risk stratification, and clinical problems. Mediterr J Hematol Infect Dis.

[12] Hunter ZR, Manning RJ, Hanzis C (2010). IgA and IgG hypogammaglobulinemia in Waldenström's macroglobulinemia. Haematologica.

[13] Vargas-Blanco DA, Hepworth OW, Basham KJ (2024). BTK inhibitor-induced defects in human neutrophil effector activity against Aspergillus fumigatus are restored by TNF-α. JCI Insight.

[14] Janniger CK, Schwartz RA, Szepietowski JC, Reich A (2005). Intertrigo and common secondary skin infections. Am Fam Physician.

[15] Thai T, Salisbury BH, Zito PM (2025). Ciprofloxacin. https://www.ncbi.nlm.nih.gov/books/NBK535454/.

